# LRRK2 in peripheral and central nervous system innate immunity: its link to Parkinson's disease

**DOI:** 10.1042/BST20160262

**Published:** 2017-02-15

**Authors:** Heyne Lee, William S. James, Sally A. Cowley

**Affiliations:** Sir William Dunn School of Pathology, University of Oxford, South Parks Road, Oxford OX1 3RE, U.K.

**Keywords:** induced pluripotent stem cells, leucine-rich repeat kinase, macrophages, microglia, neuroinflammation, Parkinson's disease

## Abstract

Mutations in the *leucine-rich repeat kinase 2* (*LRRK2*) gene are found in familial and idiopathic cases of Parkinson's disease (PD), but are also associated with immune-related disorders, notably Crohn's disease and leprosy. Although the physiological function of LRRK2 protein remains largely elusive, increasing evidence suggests that it plays a role in innate immunity, a process that also has been implicated in neurodegenerative diseases, including PD. Innate immunity involves macrophages and microglia, in which endogenous LRRK2 expression is precisely regulated and expression is strongly up-regulated upon cell activation. This brief report discusses the current understanding of the involvement of LRRK2 in innate immunity particularly in relation to PD, critically examining its role in myeloid cells, particularly macrophages and microglia.

## Parkinson's disease

Parkinson's disease (PD) is a complex, multifactorial neurodegenerative disease. In North America, it affects 1.5% of the population over the age of 65. Patients gradually develop motor impairments, caused by a slow and progressive degeneration of dopaminergic neurones in the *substantia nigra pars compacta* (SNpc). The aetiology of PD is largely unknown, involving a complex interaction between various genetic and environmental factors. To date, 17 distinctive chromosomal locations, named *parkin* (*PARK*) *1–18*, have been identified in association with inherited PD. Although only ∼10% of PD cases are identified as familial PD, genome-wide association studies (GWAS) have also detected a role for genetic variants in idiopathic PD (reviewed in ref. [1]). Understanding the roles of PD-associated genes, therefore, has increasing significance as this would provide valuable insights into shared pathological mechanisms underlying both inherited and idiopathic PD pathogenesis.

Interestingly, key PD-associated genes, *α-synuclein* (*SNCA*), *PARK2*, *deglycase* (*DJ-1*), *leucine-rich repeat kinase 2* (*LRRK2*), and *glucocerebrosidase* (*GBA*), are all expressed in immune cells, implying their potential role in immunity (reviewed in ref. [2]). Neuronal injuries commonly elicit activation of innate immune responses in the central nervous system (CNS), and inflammation-driven neurotoxicity has been suggested to play a central role in progression of various neurodegenerative diseases, including PD (reviewed in ref. [3]). PD-associated genes may have distinct cellular functions in immune cells, and it can also be hypothesized that mutations in these genes commonly contribute to abnormal immune responses, which in turn may act as a driving force to exacerbate the progression of inflammation-mediated neurodegeneration.

## Systemic and CNS inflammation in PD

The PD brain displays numerous signs of ongoing inflammatory processes. Both PD patients and animal models of PD display higher levels of activated microglia, which remain phagocytic for a prolonged period of time [4,5]. Elevated levels of inflammatory cytokines, especially tumour necrosis factor-α (TNF-α), interleukin-1β (IL-1β), IL-2, IL-6, IL-8, and interferon-γ (IFN-γ), are detected in the brain, cerebrospinal fluid, and blood of PD patients (reviewed in ref. [2]). Infiltration of peripheral immune cells, notably CD4+ and CD8+ T lymphocytes, is found as a consequence of abnormal permeability of the blood–brain barrier, which normally keeps the CNS in an immune-privileged state [6]. The involvement of humoral immunity has also been implicated: Lewy bodies and dopaminergic neurones in the SNpc show strong immunolabelling for immunoglobulin G [7]. Collectively, all of these ongoing inflammatory processes involve activation of microglia, suggesting its relevance to the pathophysiology of PD.

Microglia, the resident macrophages in the CNS, ensure a healthy environment for neurones by conducting a suite of homoeostatic functions. These include clearing cell debris, extracellular protein aggregates, and excess neurotransmitters. In response to pathological stimuli, microglia become activated, proliferate, and accumulate at the site of injury, where they phagocytose dead cells and secrete inflammatory mediators and a myriad of cytotoxic factors, especially reactive oxygen species (ROS) and nitric oxide [8]. Activation and subsequent down-regulation of microglial activity are strictly controlled, as exaggerated inflammatory responses can be harmful to neurones [9].

Chronic pathological factors (including repeated exposure to environmental toxins, genetic predispositions, and abnormal immune responses) may prolong the activated state of microglia, potentially instigating a feedforward cycle of chronic degeneration of neurones and inflammation. This self-perpetuating cycle of microglia-mediated neurotoxicity is particularly relevant in PD. First, dopaminergic neurones in the SNpc are intrinsically vulnerable to metabolic stress, particularly that caused by dopamine oxidation or mitochondrial dysfunction. High cytosolic dopamine levels can be dangerous, as dopamine metabolites (e.g. 6-hydroxydopamine) are toxic to neurones. Furthermore, compared with mesolimbic dopaminergic neurones, dopaminergic neurones in the SNpc exhibit a much larger Ca^2+^ influx, which requires the endoplasmic reticulum (ER)–mitochondrial system to clear excess Ca^2+^ (reviewed in ref. [10]). Secondly, the SNpc is one of the brain regions with the highest density of microglia and a relatively low density of astrocytes [11,12]. These factors suggest that the feedforward cycle of chronic activation of microglia and chronic damage of dopaminergic neurones would be particularly detrimental in the SNpc.

**Figure 1.**
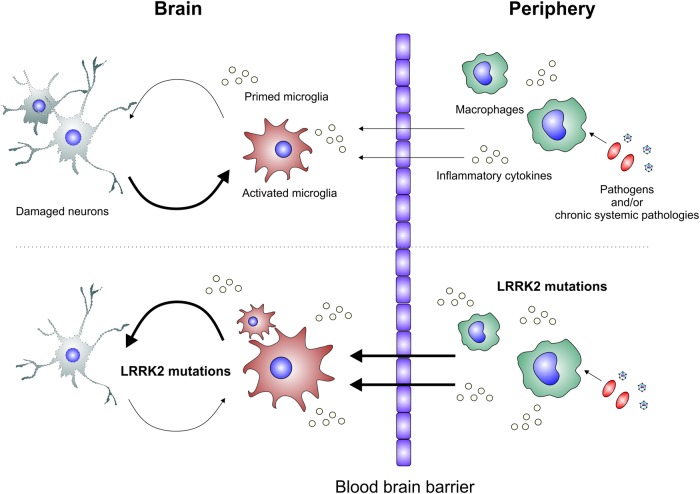
LRRK2 and microglia-driven neuroinflammation in PD. (Top panel) In this scenario, mutations in PD genes exert their effects directly in neurones, leading to chronic neuronal damage. This triggers microglial activation, leading to a vicious cycle of neuronal death and chronically activated microglia, with inflammatory cytokines causing collateral damage. The threshold for microglial activation may be lowered by peripheral inflammation, which ‘primes’ microglia. (Bottom panel) In this scenario, mutations in PD genes (in this case LRRK2) also exert effects by expression in macrophages and microglia, leading to dysfunctional immune responses. LRRK2-mutant microglia may exhibit exaggerated responses to neuronal damage, causing an amplified vicious cycle. The threshold for microglia activation could be further lowered by LRRK2 mutations within peripheral immune cells contributing to chronic microglial priming.

Systemic inflammation may also contribute to this cycle by amplifying microglia activation. The concept of microglia priming was proposed to describe exaggerated and prolonged inflammatory phenotypes displayed by microglia upon exposure to subsequent stimuli (reviewed in ref. [9]). For example, infection with *Salmonella* has been shown to activate microglia, and subsequent injection of lipopolysaccharide (LPS) to the brain triggered much higher microglial responses [13]. In the context of PD, this has been experimentally shown in rats, where prenatal exposure to LPS makes the brain more susceptible to subsequent LPS injection in midlife, leading to progressive loss of nigral dopaminergic neurones [14]. Since PD-associated genes are found in both peripheral and CNS immune cells, pathological interplay between the two immune systems carrying PD mutations could predispose the brain to reach a critical threshold of inflammation, triggering a self-perpetuating cycle of inflammation and neuronal death (Figure 1).

**Figure 2.**
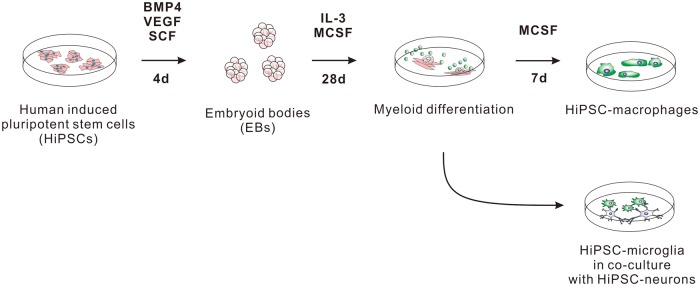
Schematic of macrophage and microglia differentiation from HiPSCs. For the detailed protocol for HiPSC-derived macrophage differentiation, see ref. [52]. BMP4, bone morphogenetic protein 4; VEGF, vascular endothelial growth factor; SCF, stem cell factor; IL-3, interleukin 3; MCSF, macrophage colony-stimulating factor; d, days.

## Leucine-rich repeat kinase 2

LRRK2 is a large, multidomain protein, displaying both GTPase and kinase activities. Most PD-causing mutations, notably R1441C/G and G2019S, cluster within these two enzymatic sites, which are surrounded by large protein–protein interacting domains (reviewed in ref. [15]). *LRRK2* mutations are one of the most common genetic causes of PD: mutations can account for as much as 40% of familial PD [16] and its variants are also found within idiopathic cases [17]. Unlike other PD-associated genes, *LRRK2* Parkinsonism manifests similar clinical phenotypes to idiopathic PD, displaying strong age-dependent development of PD symptoms [18]. Deciphering the role of LRRK2 in PD pathogenesis may reveal common pathological mechanisms underlying idiopathic PD and is consequently of great research interest.

Despite intense research effort over the past decade, the physiological function of LRRK2 and the contribution of mutations to PD remain largely elusive. This is at least in part because earlier research has mainly focussed on the role of LRRK2 in neurones, in which endogenous expression is low [19]. Many studies have, therefore, relied on overexpression of LRRK2 in non-physiologically relevant cell lines or animal models, but these approaches generate results that do not necessarily reflect the normal physiological interactome of LRRK2. This, together with the complex, multidomain structure of LRRK2, with protein–protein interaction domains at the N- and C-terminal segments, has led to LRRK2 being reported to interact with numerous molecules in a wide variety of cellular pathways, including endosome vesicle trafficking, cytoskeleton reorganization, mitochondrial function, regulation of ER/Golgi retromer complex, autophagy, and various signalling pathways, including wingless/int, TNF-α/Fas ligand (FasL)/Fas-associated protein with death domain, mitogen-activated protein kinase, and nuclear factor κ-light-chain-enhancer of activated B cells pathways [20–23]. Future investigations should examine LRRK2 expressed at physiological levels from its endogenous promoter at its normal human chromosomal location in authentic, relevant human cells, to discern which of these cellular pathways represent the bona fide function(s) of LRRK2.

## LRRK2 expression is precisely regulated in myeloid cells

Although the existence of LRRK2 protein in microglia and astrocytes has been reported in the past [24], and *LRRK2* variants have been linked through GWAS to Crohn's disease [25,26] and leprosy [27], it was not until 2010 that researchers found that LRRK2 expression is precisely up-regulated by inflammatory signals in myeloid cells, strongly implicating its potential role as a regulator of immune responses [21].

Although the level of endogenous LRRK2 is low in resting leukocytes, upon stimulation with IFN-γ, robust up-regulation of endogenous LRRK2 has been consistently detected across various subsets of myeloid cells and lymphocytes, human peripheral blood mononuclear cell-derived CD11b+ monocytes, CD3+ T lymphocytes, CD19+ B lymphocytes [21], human primary monocyte-derived macrophages, mouse primary microglia [28], and transformed cell lines, including human THP-1 monocytic leukaemia cells [29,30], and murine RAW264.7 macrophage-like cells [21]. IFN-γ activation has a direct effect on the *LRRK2* promoter region, which contains binding sites for IFN-response factors [21]. Janus kinase/signal transducers and activators of transcription and the extracellular signal-regulated kinase 5 mediate IFN-γ-induced LRRK2 up-regulation in macrophages, although the exact signalling cascades are yet to be elucidated [29].

LRRK2 is also moderately inducible by other inflammatory mediators, namely IFN-β, TNF-α, and IL-6 [31], whereas the Toll-like receptor 4 (TLR4) agonist, LPS, is found to have an inconsistent effect. Some groups have reported significant up-regulation of LRRK2 protein expression by LPS in primary mouse microglia or in THP-1 cells [28,32], whereas others did not detect any changes in murine immortalised microglia (BV-2), primary mouse microglia [33,34], or mouse bone marrow-derived macrophages (BMDMs) [35]. This discrepancy could be attributed to many factors, such as cell types, experimental conditions, or technical variations. Regardless, activation of TLR4 reproducibly leads to phosphorylation at Ser910/935 residues of LRRK2 in all myeloid cell lineages [19,35,36]. Phosphorylation at Ser910/935 determines its cellular localization, interaction with 14-3-3 protein, dimerization, and translocation from cytosol to the membrane [19,37–39]. However, further experimental investigation is needed to understand the direct physiological consequences of phosphorylation at Ser910/935 residues in myeloid cells.

## Roles of LRRK2 in macrophages and microglia

Microglia and macrophages are both classified as mononuclear phagocytes, sharing common functions of various maintenance and protective roles, but can be distinguished by their ontogeny and transcription profiles (reviewed in ref. [40]). Since biochemical changes in LRRK2 upon inflammatory cues are identical in both systems [19], efforts have been made to inspect the role of LRRK2 in various aspects of innate immunity, summarized in Table 1. These studies imply opposing roles of LRRK2 in peripheral and CNS innate immunity. However, it should be noted that none of these studies has directly compared microglia and macrophages under the same experimental conditions, so direct evidence for opposing roles is still lacking. Moreover, most data are from mouse models that do not faithfully recapitulate all aspects of the human immune system [41].

**Table 1:** Summary of reports on the role of LRRK2 in innate immunity

Species	Cell types	Methods	Results	References
LPS-mediated cytokine and chemokine release				
Mouse	BV-2	LRRK2 knockdown (KD) shRNA	↓TNF-α, IL-6, Nitrite	[42]
Mouse	Primary microglia	LRRK2 KD RNAi Kinase inhibition: sunitinib LRRK2-in-1	↓TNF-α	[32]
Mouse	Primary microglia	LRRK2 KO Kinase inhibition: LRRK2-in-1 GSK2578215A	↓IL-1β, cyclooxygenase-2 mRNA	[33]
Mouse	Primary microglia	R1441G	↑TNF-α; ↓IL-10	[28]
Mouse	BMDMs	R1441C LRRK2 KO	No difference in IL-6 or keratinocyte chemokine (KC)	[43]
Mouse	BMDMs	LRRK2 KO	No difference in TNF-α, IL-6, KC, IL-1β, IL-10, IL-12	[35]
Mouse	Thioglycollate-elicited peritoneal macrophages (TEPMs)	LRRK2 KO	No difference in IL-1β, IL-10, IL-1α, TNF-α, IL-6, KC, granulocyte colony-stimulating factor, monocyte chemoattractant protein-1	[44]
Mouse	TEPMs	G2019S	No difference in TNF-α	[36]
Migration				
Mouse	Primary microglia BV-2	G2019S	↓ADP-induced migration	[48]
		LRRK2 KD shRNA	↑ADP-induced migration	
Mouse	Primary microglia	LRRK2 KO	↑Fractalkine-induced migration	[49]
Mouse	Primary TEPMs	G2019S	↑ADP-induced migration	[36]
		Kinase inhibition SRI 29451 HG-10-102-01	↓G2019S-enhanced ADP-induced migration	
Phagocytosis				
Mouse	BV-2 RAW264.7	LRRK2 KD shRNA Kinase inhibition LRRK2-in-1 GSK2578215A HG-10-102-01	No difference in uptake of FITC-conjugated beads	[19]
Mouse	Primary TEPMs	G2019S	No difference in uptake of fluorescent zymosan bioparticles	[36]
Mouse	RAW264.7	*Salmonella typhimurium* infection	LRRK2 localization to phagosomes	[21]
		LRRK2 KD SiRNA	↓ROS with zymosan ↑Survival of intracellular *S. typhimurium*	

### Cytokine release

In mouse primary microglia, R1441G mutation leads to an increase in LPS-driven inflammatory cytokine release [28], and abolishing LRRK2 protein expression has the opposite effect [32,33,42]. However, in mouse primary macrophages, neither R1441G [43] nor G2019S mutations [36], nor *LRRK2* knockout (KO) [35,43,44], cause any change in LPS-driven cytokine release. Only Dectin-1 activation by zymosan (yeast) has been shown to produce higher levels of cytokine release in *LRRK2* KO mouse macrophages [45]. Therefore, in macrophages, LRRK2 may be dispensable in TLR4-mediated cytokine release, but may serve an important role in responding to other inflammatory stimuli. A recent report has shown that higher levels of peripheral inflammatory cytokines were found in the sera of both asymptomatic LRRK2 G2019S carriers and PD patients carrying LRRK2 G2019S [46], suggesting pathological contributions from *LRRK2* mutations within peripheral immune cells. Further studies are merited to establish which specific inflammatory pathways are mediated by LRRK2 in macrophages or microglia. Additionally, the role of LRRK2 in peripheral cytokine release is still equivocal, and it will help the field to have studies that directly compare cytokine release in microglia and macrophages from the same animals under the same experimental conditions, comparing all the key *LRRK2* mutations and KO together. Finally, LRRK2-mediated immune responses by more disease-relevant immunogenic agonists, such as aggregates of SNCA, should be explored further, as there is evidence for close interaction between α-synuclein and LRRK2 (reviewed in ref. [47]).

### Migration

The ability to respond and migrate to the site of injury or infection is a key feature of innate immunity, and recent evidence suggests the involvement of LRRK2 in this process. Abolishing LRRK2 expression in BV-2 microglial-like cells [48] or mouse primary microglia [49] leads to significantly higher migration versus wild type, and the G2019S mutation in mouse primary microglia diminishes ADP-induced migration [48]. However, in mouse primary macrophages, the same mutation has been shown to have the opposite effect, enhancing motility towards ADP [36].

### Phagocytosis

The absence of LRRK2 activity in BV-2 microglial-like cells or RAW 264.7 macrophage-like cells has been reported to have no effect on phagocytosis [19]; likewise, G2019S mutation was not observed in mouse primary macrophages [36]. However, it has been reported that LRRK2 localizes to phagosomes upon bacterial infection, and that lack of LRRK2 expression reduces ROS production and enhances bacterial survival in RAW 264.7 cells [21]. Therefore, LRRK2 may have a specific role during phagocytosis, which may not be detected by simple phagocytosis assays measuring initial uptake of bioparticles. Indeed, LRRK2 is implicated in autophagy in myeloid cells ([19]; reviewed in ref. [50]), a pathway that shares common features with phagocytosis and is also involved in innate immunity (reviewed in ref. [51]). Further exploration of the role of LRRK2 during specific stages of phagocytosis pathways is merited.

## Human-induced pluripotent stem cell macrophages and microglia as tools to study LRRK2 biology

It is clear from above that the data on the role of LRRK2 in myeloid cells has so far been collected primarily from murine *ex vivo* models or from transformed murine cell lines, under a wide variety of experimental conditions. While murine systems are extremely useful, they do not precisely replicate all human cellular and biochemical pathways [41]. Moreover, transformed cell lines poorly reproduce the cellular physiology of authentic primary human macrophages and microglia, which are terminally differentiated cells. Human-induced pluripotent stem cell (HiPSC)-derived macrophages provide an attractive and highly authentic model to study LRRK2 biology (Figure 2). HiPSC-derived macrophages are genetically tractable, can be generated efficiently, and, at scale, become terminally differentiated and accurately recapitulate macrophage functionality [52]. Together with clustered regularly interspaced short palindromic repeats (CRISPR)/Cas-9 gene editing of iPSCs, one can investigate LRRK2 protein at the endogenous level by generating KO lines, correcting and introducing mutations, creating reporter lines, or tagging endogenous proteins. The applicability of this system has been shown already in other diseases, including HIV and chronic granulomatous disease [53,54]. Methods for skewing HiPSC-derived macrophages to microglia are currently under development and will enable direct comparisons of LRRK2 function in human macrophages and microglia.

## Concluding remarks

Recent evidence supports the idea that pathological interplay between peripheral and CNS innate immunity probably contributes to the progression of PD. LRRK2 may be involved in this interplay, as expression of LRRK2 is tightly regulated in both systems and evidence reviewed here implicates LRRK2 in both peripheral and CNS innate immunity. Although the current literature appears to suggest that LRRK2 plays distinct roles in microglia and macrophages, more work needs to be done to unequivocally establish the *bona fide* function(s) of LRRK2 in human macrophages and microglia, and the role of *LRRK2* mutations in these cells in PD. To achieve this, macrophages/microglia differentiated from HiPSCs provide a powerful tool to better understand LRRK2-mediated pathology in PD and also other LRRK2-mediated immune disorders.

## Abbreviations

BMDMs: bone marrow-derived macrophages
CNS: central nervous system
CRISPR: clustered regularly interspaced short palindromic repeats
ER: endoplasmic reticulum
FasL: Fas ligand
GWAS: genome-wide association studies
HiPSC: human-induced pluripotent stem cell
IFN-γ: interferon-γ
IL-1β: interleukin-1β
KO: knockout
LPS: lipopolysaccharide
*LRRK2*: leucine-rich repeat kinase 2
PARK2: *parkin*
PBMC: peripheral blood mononuclear cell
PD: Parkinson's disease
ROS: reactive oxygen species
*SNCA*: *α-synuclein*
SNpc: *substantia nigra pars compacta*
TLR: Toll-like receptor
TNF-α: tumour necrosis factor-α.
